# Tracing the Attention of Moving Citizens

**DOI:** 10.1038/srep33103

**Published:** 2016-09-09

**Authors:** Lingfei Wu, Cheng-Jun Wang

**Affiliations:** 1 Knowledge Lab, Computation Institute, University of Chicago, Chicago, IL., 60607, United States; 2 Computational Communication Collaboratory, School of Journalism and Communication, Nanjing University, Nanjing, 210093, P. R. China

## Abstract

With the widespread use of mobile computing devices in contemporary society, our trajectories in the physical space and virtual world are increasingly closely connected. Using the anonymous smartphone data of 1 × 10^5^ users in a major city of China, we study the interplay between online and offline human behaviors by constructing the mobility network (offline) and the attention network (online). Using the network renormalization technique, we find that they belong to two different classes: the mobility network is small-world, whereas the attention network is fractal. We then divide the city into different areas based on the features of the mobility network discovered under renormalization. Interestingly, this spatial division manifests the location-based online behaviors, for example shopping, dating, and taxi-requesting. Finally, we offer a geometric network model to help us understand the relationship between small-world and fractal networks.

Digital media, especially Internet and smartphones, provides a new lens to study human behaviors in both the physical space and the virtual world. Researchers are getting more interested in studying the relationship between online and offline human behaviors^1^,^2^,^3^. For example, Ginsberg *et al*. tried to predict the influenza epidemics using search engine query data^1^; Bond *et al*. studied the Facebook usage on offline political mobilization^2^; Zhao *et al*. confirmed the correlation between human movements in cyberspace and physical space using a mobile phone dataset^3^. However, the differences of online behaviors in different physical areas remain unexplored. In this study, we tackle this problem using high-resolution smartphone data of 1 × 10^5^ users in a major city of China.

We construct a mobility network (offline) and an attention network (online) to compare their structures. In the mobility network, nodes are base stations and edges represent the movements of users between base stations^4^,^5^, while in the attention network, nodes are websites and edges represent the “hops” of users between websites^6^. Prior studies showed that using the renormalization method one can classify networks into two universal classes: the small-world networks and the fractal networks^7^,^8^. In small-world networks, the total number of nodes *N* increases exponentially with the average diameter of the network *l*, that is, 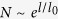 (where *l*_0_ is a characteristic length), whereas the fractal property requires a power-law relation between *N* and *l*, that is, *N* ~ *l*^−*α*^. Using the box-covering method proposed by Song *et al*.^7^, we find that the online and offline networks belong to different classes: the attention network exhibits fractal properties, whereas the mobility network is small-world.

We also find that the small-world feature of the mobility network fades out when we decrease the resolution of the network by aggregating base stations near to each other into “supernodes”^7^,^8^. In particular, the positive degree correlation, which is suggested to be the signature of the small-world pattern^8^, turns into negative when we aggregate nodes together. We use the box-covering method to renormalize the network and the transition of degree correlations occurs when the box length *l*_*B*_ goes beyond 3.

A closer look at the network reveals that high degree nodes (base stations) form spatially distant clusters in which they are tightly connected to each other. In the renormalization process, when clusters are collapsed into “supernodes”^7^,^8^, the connections between high degree nodes within the same cluster disappear. Therefore, the transition point *l*_*B*_ = 4 implies the characteristic size of spatially distant clusters. Based on this observation, we divide the city into different areas by renormalizing the network with *l*_*B*_ = 4 and identify the top websites in each area. We present three examples of location-based online behaviors, including shopping, dating and taxi-requesting. Finally, we offer a geometric network model which sheds light on the relationship between small-world networks and fractal networks^9^.

## Results

### Two Universal Classes of Behaviors

**Figure 1:**
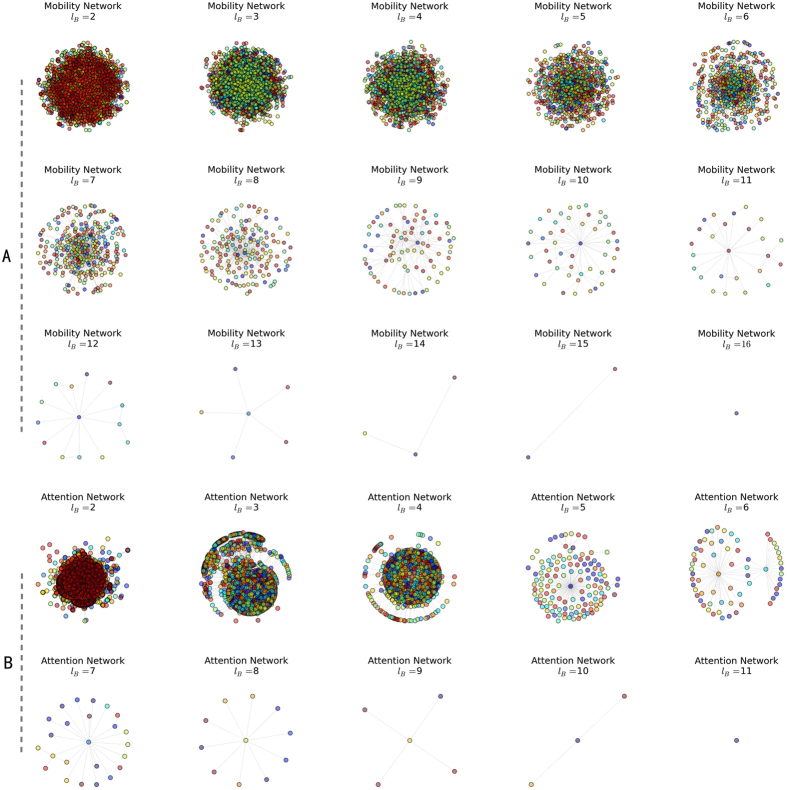
Renormalization steps of the mobility network (**A**) and attention network (**B**). It takes 15 and 10 steps to tile the two networks, respectively.

Song *et al*. revealed the fractal property of complex networks using the box-counting method^7^. We apply the same renormalization technique in both the mobility network and the attention network. Figure 1 shows the renormalization steps for two networks. There are 9,899 nodes and 39,083 edges in the mobility network, and 16,476 nodes and 144,909 edges in the attention network. The diameter of the mobility network and the attention network is 15 and 10, respectively. The length of diameter also indicates the steps needed to collapse the networks into a single node.

**Figure 2:**
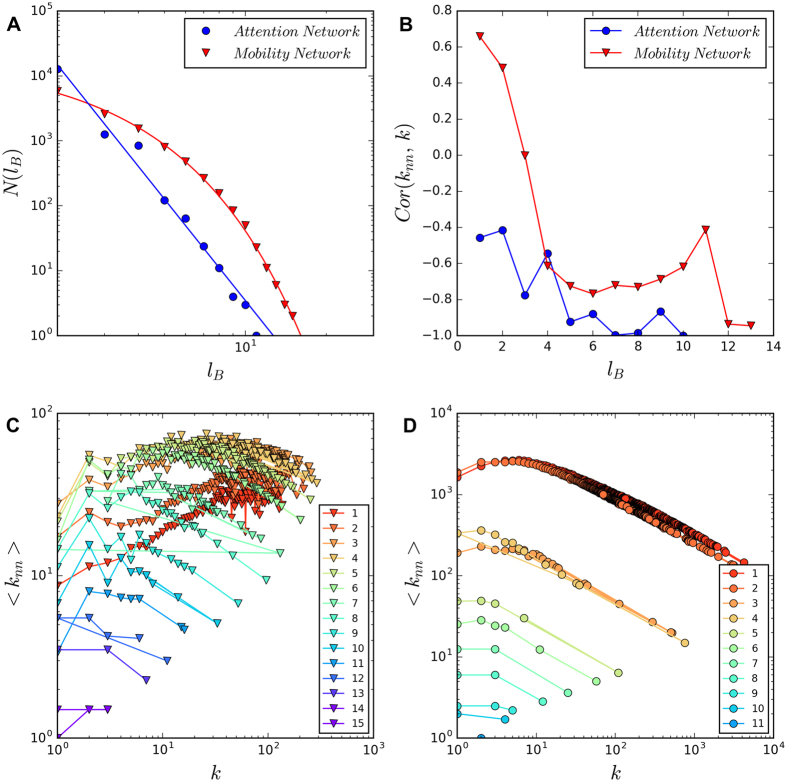
Two universal classes of behaviors and the transition of degree correlations. (**A**) The number of boxes *N*(*l*_*B*_) is a power law function of box length *l*_*B*_ in the attention network, and these two variables show an exponential relationship in the mobility network. (**B**) In the mobility network, the degree correlation (measured by the Pearson correlation coefficient *Cor*(*k*_*nn*_, *k*)) decreases from positive to negative when *l*_*B*_ = 4, while the correlation remains negative in the attention network. Panel (C,D) show the transition of degree correlation in details. For the mobility network (**C**), the slope of data points is positive when 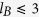, and the slope turns negative when 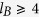. Meanwhile, the correlation is always negative in the attention network (**D**).

The online and offline networks show very different properties in the renormalization process (Fig. 2B). In the attention network, the number of boxes *N*(*l*_*B*_) scales to the length of box *l*_*B*_, following a power-law function, 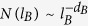. However, in the mobility network, *N*(*l*_*B*_) and *l*_*B*_ display an exponential relationship *N*(*l*_*B*_) ~ *e*^−*β*^. According to Song *et al*.^7^, the mobility network is small-world and the attention network is fractal. This findings imply that online and offline human behaviors are governed by different mechanisms.

### Transition of Degree Correlations

The degree correlation of a network *Cor*(*k*_*nn*_, *k*) measures how the average degree of neighbors <*k*_*nn*_> changes with the degree k of nodes^10^. It is suggested that the small-world property is relevant to positive degree correlation, i.e., high degree nodes tend to connect to each other; and the fractal property is relevant to negative degree correlation^8^. We confirm this result in Fig. 2. Interestingly, we find that the positive degree correlation in the mobility network changes into negative with the increase of *l*_*B*_. In particular, *Cor*(*k*_*nn*_, *k*) > 0 when *l*_*B*_ < 4, and *Cor* (*k*_*nn*_, *k*) < 0 when 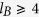.

This is because the nodes of the mobility network, i.e., the base stations, are distributed very unevenly in the city. Since the spatial distribution of population is nonuniform, more base stations have to be set up to provide services in the areas of high population density. Meanwhile, as confirmed in previous research^11^,^12^, most individuals move frequently over short distances and rarely across long distances. As a consequence, local clusters of base stations containing high degree nodes connected to each other emerge from the daily transit activities of residents. This explains the observed positive degree correlations. In the process of renormalization, these local clusters are collapsed into “supernodes” at a characteristic scale (e.g., *l*_*B*_ = 4). These “supernodes” (local clusters of base stations) under renormalization are still high in degree (because they are also connected to many base stations outside the cluster), but they are not close to each other, neither do they connect to each other directly. Therefore, the degree correlation becomes negative in the renormalized network when nodes are aggregated. To sum up, by analyzing the critical point at which degree correlation shifts from positive to negative, we are able to reveal the average, characteristic scale of high population density regions in the city. This paves the way for investigation of location-based behaviors.

### Location-Based Human Behaviors

**Figure 3:**
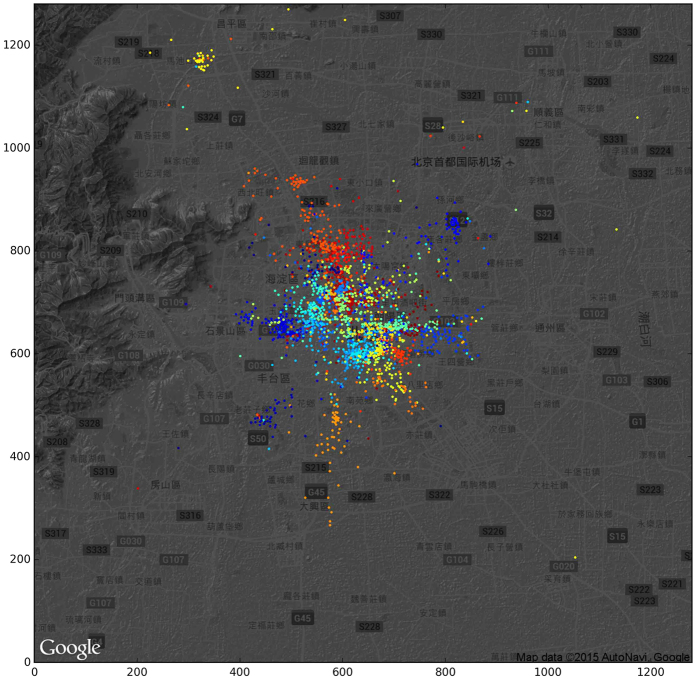
Community detection of the mobility network. The top 20 largest communities are visualized with different colors. Base map used in this figure is from Google Map.

The aforementioned analysis shows that the box-covering technique can help separate the entire city (mobility network) into different regions (communities) containing intensive local traffic when *l*_*B*_ = 4 (see Fig. 3). It would be interesting to investigate the social-functional difference of these regions, i.e., whether users have different online behaviors across regions.

**Figure 4:**
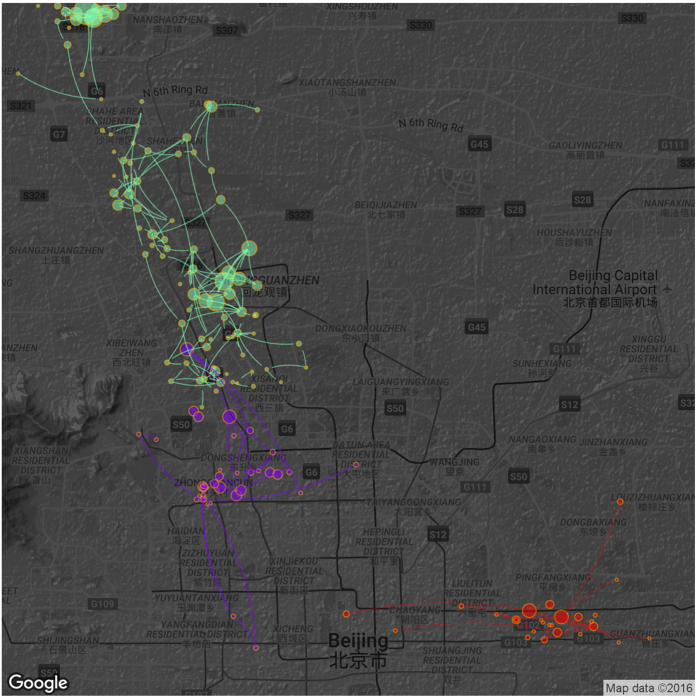
Three example communities in the mobility network, including shopping (red nodes and edges), dating (purple nodes and edges), and Taxi-requesting (green nodes and edges). Base map used in this figure is from Google Map.

For each region, we identify the most relevant websites using the TF-IDF method. TF-IDF is a measurement widely used to find the important words for each document in natural language processing. In our case, we treat websites as terms, the detected regions (from which the visits to websites are generated) as documents. Then we calculate the TF-IDF indices of websites within each region in order to identify the most relevant websites. These websites provide clues to infer the location-based behaviors. We present the results of three regions in Fig. 4: A. Shopping (the red nodes and edges). This region belongs to the Chaoyang district. It covers the Guomao road, around which is one of the largest business circles of Beijing. B. Dating (the purple nodes and edges). This region belongs to the Haidian district, where several top universities in China are located, including Peking University, Tsinghua University, and Renmin University. C. Taxi-requesting (the green nodes and edges). This narrow region connects downtown with Changping District in the north-west of Beijing. This area is notorious for high commuting traffic and inefficient public transit, especially before a new subway line was opened in late 2015 (the analyzed data are collected before 2014).

### Geometric Network Models

Zhang *et al*. proposed a growing geometric graph model to model the growth of cities^13^. Initially a seed node is placed at the center of a d-dimensional Euclidean space. At each time step a new node is generated randomly within the space, added to the graph, and connected to all existing nodes within distance r. If there are no existing nodes within r, this new node will be removed. We call this version “Model-all” and change the linking rule to develop two versions, including 1). the new nodes are connected to the highest-degree node within distance r (Model-max), and 2). the new nodes are connected to the lowest-degree node within distance r (Model-min).

**Figure 5:**
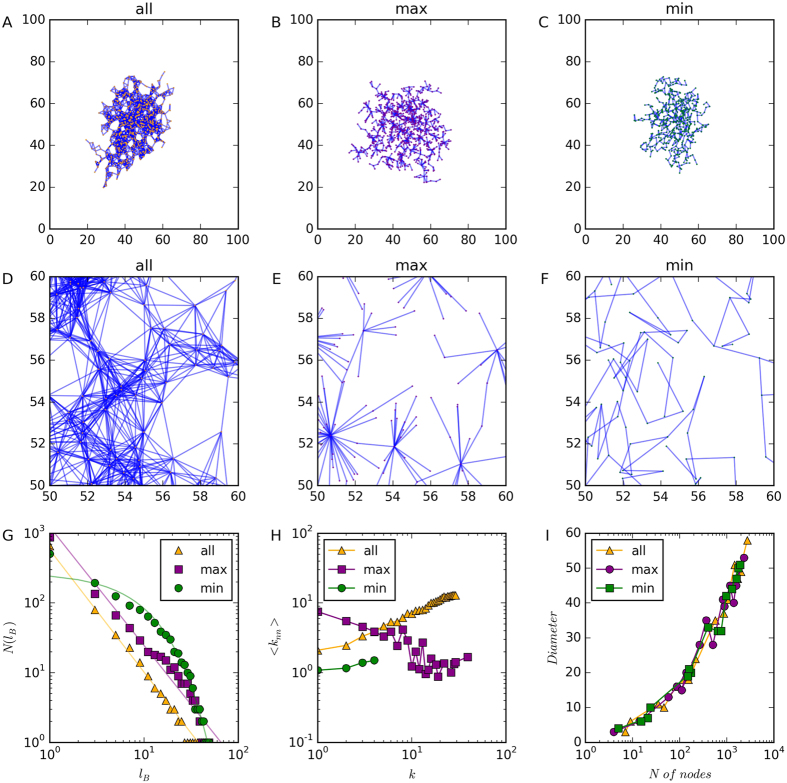
Geometric network models. The first column shows the model proposed by Zhang *et al*. (Model-all)^13^, in which a new node is connected to all nodes within a fixed radius r. The second and the third columns show the other two versions of models, in which a new node is connected to the highest degree node (Model-max) and the lowest degree node (Model-min), respectively. The first row shows the global structure of networks and the second row shows a local part of the networks. The parameters of the model are set as follows: the size of 2D space = 100 × 100, the radius = 3, the number of simulation time steps = 10,000. The third row compares the dynamics of different models and shows that Model-min and Model-max replicates the patterns observed in the mobility network (positive degree correlation and exponential relation between *N*(*l*_*B*_) and *l*_*B*_) and in the attention network (negative degree correlation and power-law relation between *N*(*l*_*B*_) and *l*_*B*_), respectively.

We find that Model-min shows positive degree correlation and Model-max displays negative degree correlation (Fig. 5H). Meanwhile, the number of box *N*(*l*_*B*_) is a power-law function of the length of box *l*_*B*_ in Model-max, whereas it is an exponential function of *l*_*B*_ in Model-min (Fig. 5G). Therefore, Model-min and Model-max replicate the patterns observed in the mobility network and in the attention network, respectively.

The comparisons between the aforementioned models provide insights into our understanding of human behaviors in physical and virtual worlds. As shown in previous research^3^, both the browsing behavior and the physical movement of human are governed by two underlying driven forces, exploration of new sites and preferential return to highly visited sites. However, the exploration tendency is much stronger in the virtual world than that in the physical space. Therefore, with a high probability users hop between popular and non-popular websites, connecting high degree nodes with low degree ones. In contrast, in the physical world users preferentially return to living and/or working places for most of time. However, to get back to the preferred place, one has to go through a sequence of consecutive base stations in the local clusters. Thus, whenever users are visiting a new base station that is seldom visited (a low degree node) during a long trip across the city, it is very likely that they will pass another less frequently visited base station. Our models capture the discussed differences between offline and online behavior, thus successfully replicate the observed properties of the mobility and attention networks.

## Discussion

This research compares online and offline human behaviors by constructing two networks, the attention network and the mobility network. The attention network is fractal, in which high degree nodes tend to connect to low degree nodes; whereas the mobility network is a small-world network, in which high degree nodes are connected with each other. The structural difference may hint on different organizing principles of human behaviors in online and offline space, e.g., the small-world structure of the mobility network has the advantage of efficient transportation^14^, whereas the fractal character of the attention network tends to create a hierarchy of self-similar nested modules^8^, and to increase the network robustness^8^. More interestingly, we observe the transition of degree correlations in the mobility network, which reveals the spatially constrained human behaviors, for example, shopping, dating, and taxi-requesting. Finally, we use a geometric network model to test our assumptions on the origins of the observed fractal and small-world patterns in individual behaviors. The spatial-constrained attachment of the geometric network models indicates that online and offline human behaviors follow a general principle of locality (two close entities have higher probabilities to be connected)^13^, but their linking dynamics are different. A highlight of the current research is that while previous research mainly focuses on the common patterns shared by online and offline behaviors^3^, we suggest that online and offline behavior could be governed by very different mechanisms.

Our analysis on location-based behavior is not only theoretically interesting, but also has strong applied consequences. For example, we addressed an important issue in the advertising industry, that is, how to synchronize online and offline channels to deliver integrated information of product brands. The increasing power of search engines has made advertising more and more precise. Computational advertising systems like Google AdSense collect and filter the attention of the most relevant users and sale it to companies. The telecommunications operators have the most detailed information about the user’s geographical locations and the websites they are visiting. A city can be separated into functional areas using the network renormalization method. The findings about how human move in both cyberspace and physical space can help find the best spot to place online and offline advertisements or build more accurate recommendation systems.

**Figure 6:**
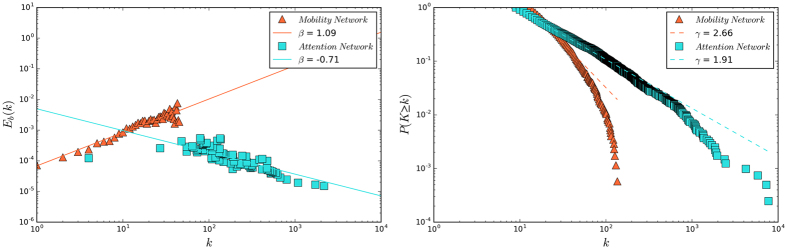
The relationship between *E*_*b*_(*k*) and degree *k* and the degree distributions. (**A**) The plot of *E*_*b*_(*k*) versus *k* for the mobility network (*β* = 1.09) and the attention network (*β* = −0.71). (**B**) The degree distributions of the mobility network (*γ* = 2.66) and the mobility network (*γ* = 1.91).

More indicators characterizing the fractal and small-world properties should be explored in further research. For example, a recent study by Gallos *et al*. proposed an alternative measure *ε* on the fractal property of networks^15^. They defined *E*_*b*_(*k*) to quantify the fraction of links from *k*-degree nodes to *bk*-degree nodes, in which *b* is trivial constant parameter. In scale-free networks the *E*_*b*_(*k*) scales to *k*, allowing the estimation of *β* in the scaling relationship *E*_*b*_(*k*) ~ *k*^*β*^. After obtaining *β*, *ε* can be calculated as *ε* = *γ* − *β*, in which *γ* is the exponent of the power-law degree distribution. Gallos *et al*. classify scale-free networks into three regions, including fractal (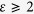), random (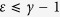), and transition (*γ* − 1 < *ε* < 2). In Fig. 6 we apply their method and calculate that *β*_*mobility*_ = 1.09, *γ*_*mobility*_ = 2.66, *β*_*attention*_ = −0.71, and *γ*_*attention*_ = 1.9. Thus, the *ε* of the two networks are 1.57 and 2.62, respectively. According to Gallos *et al*.^15^, the mobility network lies in the random region and the attention network is fractal. This conclusion is consistent with our findings.

## Methods

### Data

In this study, we primarily work on a high resolution, anonymous smartphone dataset collected from a random sample of 1 × 10^5^ users in a major city of China. The dataset includes all the websites visited by these users in a day, as well as the base stations that are providing the access to the Internet. The dataset is anonymized, and we do not have the personal information of these users.

**Figure 7:**
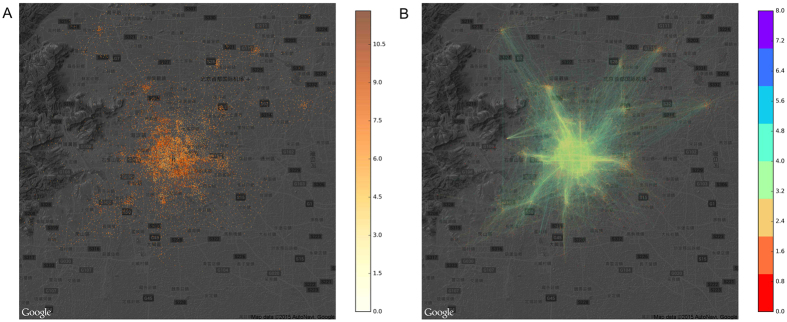
The spatial distribution of base stations and their connections (the movement of users between base stations). (**A**) The distribution of 14,909 base stations in our dataset. The brightness of the data points is proportional to the mobility traffic to these stations. (**B**) The movement of users between stations. Base map is from Google Map.

We construct two networks to trace how users move in the virtual world (attention network) and in the physical world (mobility network). In the mobility network, the nodes are mobile base stations (N = 9,899), and the edges (N = 39,083) show how users travel from one base station to another. In the attention network, the nodes (N = 16,476) are websites, and edges (E = 144,909) represent the switch of users between websites. As shown in Fig. 7A, the distribution of the base stations is uneven, as more stations are needed in high population density areas. Figure 7B shows the mobility network, which portrays the collective moving trajectory of city residents.

**Figure 8:**
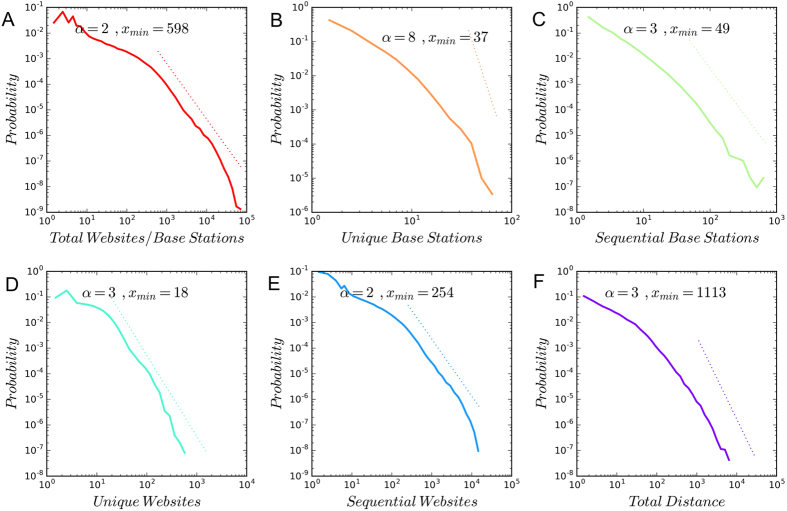
Long-tail distributions of various spatial/virtual behaviors.

**Figure 9:**
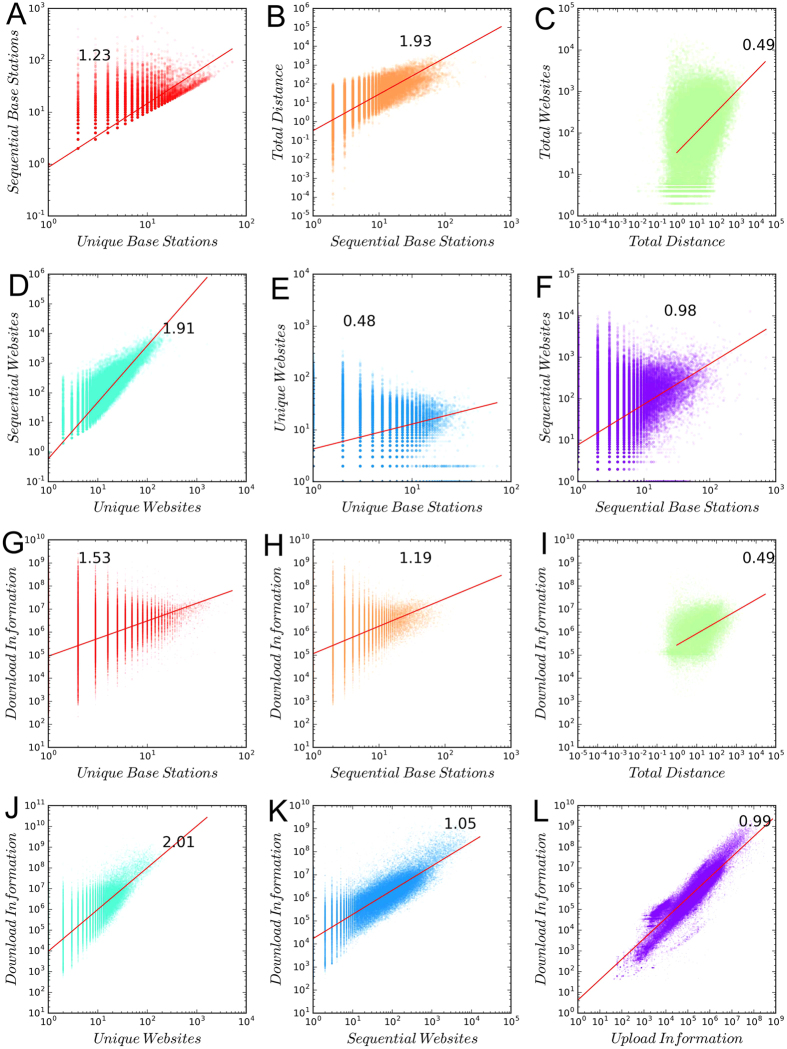
The scaling between virtual and spatial movements.

Figure 8 shows the distribution of the following six variables characterizing the behavior of users: 1) the total number of browsing requests (sent to a website from a base station); 2) the number of unique base stations; 3) the number of sequentially visited base stations (in which the successive visits to the same base stations are aggregated); 4) the total distance of the daily trips in kilometers; 5) the number of unique websites; 6) the number of sequentially visited websites. It turns out that the probability distributions of all these variables are long-tail. We fit these distributions using power law models^16^, and show the fitted exponents. To conclude, both online and offline activities are very heterogeneous among users. To investigate the correlation between online and offline behaviors. We plot various variables on online behaviors again the measures of offline activities in Fig. 9. We find that heavy users of the Web are also active offline. This is consistent with the previous research^3^. Yet, it does not mean that online and offline human behaviors are similar.

### Network Renormalization

We use the box-counting method to renormalize the two networks^7^. In each step of the renormalization process, a network is covered with boxes of length *l*_*B*_ such that the nodes within the same box are at a distance 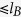 from each other. The nodes within the same box are merged into a “supernode”, and the edges between them disappear. With the increasing of *l*_*B*_, we need fewer boxes *N*(*l*_*B*_) to cover the network. These process is repeated until there all nodes are packed into the same single box.

### TF-IDF

We use the term frequency-inverse document frequency (TF-IDF) index to measure the relevance of websites. TF-IDF, which is the product of two statistics, term frequency (TF) and inverse document frequency (IDF), is widely used to measure the importance of a term to a document. TF measures the frequency of a term in a given document and document frequency (DF) measures the concentration of terms across available documents. If a term appears many times in a documents, but is rare in the rest of documents, it will have high TF and low DF (or high IDF). Therefore, the higher the TF-IDF is, the more relevant the term is to the document. In our case, we treat websites as terms, the detected regions from which the visits to websites are generated as documents, and then calculate the TF-IDF indices of websites within each region, in order to identify the most relevant websites in a region.

## Additional Information

**How to cite this article**: Wu, L. and Wang, C.-J. Tracing the Attention of Moving Citizens. *Sci. Rep.* **6**, 33103; doi: 10.1038/srep33103 (2016).
